# A Snapshot of Functional Genetic Studies in *Medicago truncatula*

**DOI:** 10.3389/fpls.2016.01175

**Published:** 2016-08-09

**Authors:** Yun Kang, Minguye Li, Senjuti Sinharoy, Jerome Verdier

**Affiliations:** ^1^Plant Biology Division, The Samuel Roberts Noble FoundationArdmore, OK, USA; ^2^University of Chinese Academy of SciencesBeijing, China; ^3^Shanghai Plant Stress Center, Shanghai Institutes of Biological Sciences, Chinese Academy of SciencesShanghai, China; ^4^Department of Biotechnology, University of CalcuttaCalcutta, India

**Keywords:** *Medicago truncatula*, functional genomics, seed, symbiosis, abiotic stresses

## Abstract

In the current context of food security, increase of plant protein production in a sustainable manner represents one of the major challenges of agronomic research, which could be partially resolved by increased cultivation of legume crops. *Medicago truncatula* is now a well-established model for legume genomic and genetic studies. With the establishment of genomics tools and mutant populations in *M. truncatula*, it has become an important resource to answer some of the basic biological questions related to plant development and stress tolerance. This review has an objective to overview a decade of genetic studies in this model plant from generation of mutant populations to nowadays. To date, the three biological fields, which have been extensively studied in *M. truncatula*, are the symbiotic nitrogen fixation, the seed development, and the abiotic stress tolerance, due to their significant agronomic impacts. In this review, we summarize functional genetic studies related to these three major biological fields. We integrated analyses of a nearly exhaustive list of genes into their biological contexts in order to provide an overview of the forefront research advances in this important legume model plant.

## Introduction

Medicago, a Mediterranean origin species, has been intensively used as a legume pasture plants worldwide. Nowadays, Alfalfa (*Medicago sativa*) is the most cultivated forage plants in the USA and represents the most economically valuable forage for animal feed. In the past 20 years, the increasing number of research projects on legumes allowed the emergence of model plants for legume species. Crop and pasture legumes are generally poor model systems for genetic and genomic research. Some cultivated legumes are tetraploid (e.g., peanut), many have large genomes (e.g., pea and faba beans) and many are recalcitrant to transformation or difficult to regenerate (e.g., common bean, pea, and soybean). Most grain legumes have large but relatively few seeds per plant, and large seedlings, which prevents high-density culture (e.g., chickpea, black-eyed pea, mung bean, pea, bean, and soybean). Some legumes, such as soybean, have genome duplications, and some are self-incompatible or have a long generation time. As a result, two species, *Medicago truncatula* and *Lotus japonicus* have been proposed as models for legume research (Barker et al., 1990; Handberg and Stougaard, 1992). *M. truncatula* was first proposed as a model by Barker et al. (1990) to study the rhizobia-legume symbiosis. Now, it is internationally recognized as a model legume for all the legume studies. *M. truncatula* has several advantages for plant genomic research: diploid genome (2n = 16), autogamous, relatively small genome (~375 Mbp), which was sequenced and annotated (Young et al., 2011), and a relatively short generation time (around 4 months seed to seed).

Dedicated meetings and workshops on Medicago have allowed a rapid and coordinated development of genetic and genomic tools. For instance, transcriptomics studies have been facilitated by the development of microarray chips such as the 16k microarray of 70-mer oligos used in studies such as Hohnjec et al. (2005) or Gallardo et al. (2007) and Affymetrix GeneChip used in studies such as Benedito et al. (2008), Verdier et al. (2013b), and Zhang et al. (2014). Most of the data obtained from the Affymetrix GeneChip experiments have been stored and publicly shared on a dedicated webserver to provide a *M. truncatula* Gene Expression atlas (MtGEA, www.mtgea.noble.org; He et al., 2009). Transcriptomics tools also comprise a high-throughput quantitative PCR platform to profile all known transcription factors used in studies such as Verdier et al. (2008). Recently, the development of RNA-seq technologies has allowed a comprehensive identification and quantification of transcripts in *M. truncatula* such those responding to different stresses (e.g., Gruber et al., 2009; Li et al., 2009; Zhang et al., 2014). In parallel to transcriptomics tools, *M. truncatula* also has libraries for metabolomics studies (Broeckling et al., 2004) and reference maps for proteomics studies (Mathesius et al., 2001; Gallardo et al., 2003; Watson et al., 2003). Recently, 330 *M. truncatula* accessions from a germplasm collection were sequenced and have been used for genome-wide association studies such as Stanton-Geddes et al. (2013) and Kang et al. (2015).

Numerous bioinformatics resources are also available for Medicago, some have been developed specifically for Medicago (and legumes) such as the Medicago Gbrowser (http://gb.sc.noble.org/cgi-bin/gb2/gbrowse), LegumeGRN (Wang M. et al., 2013), legumeIP (Li et al., 2012), and Legoo (http://www.legoo.org); and others have been adapted from Arabidopsis to Medicago such as PathExpress (Goffard and Weiller, 2007) and AgriGO (Du et al., 2010). Another key step in the adoption of *M. truncatula* as a model plant for legume studies was the possibility of *Agrobacterium*-mediated transformation of the whole plant via somatic embryogenesis using *Agrobacterium tumefaciens* (Thomas et al., 1992), transformation of seedling using *Agrobacterium tumefaciens* (Trieu et al., 2000) or more specifically transformation of roots to generate transient hairy root transformants using *Agrobacterium rhizogenes* (Boisson-Dernier et al., 2001). The emergence of functional genetics in *M. truncatula* has been possible due to its capacity to be transformed and more recently due to the generation of different mutant populations.

## Medicago mutant populations

Mutant populations play a central role in functional genomics analyses and are used in both forward and reverse genetic studies. To date, the three largest mutant populations of *M. truncatula* have been produced by three different approaches: chemical mutagenesis using Ethyl Methane Sulfonate (EMS population), fast neutron bombardment (FNB population), and finally transposon tagging with the introduction of the *Tnt1* transposon of Tobacco within the *M. truncatula* genome (*Tnt1* population). Despite that these populations are the largest and the most popular according to the number of mutated plants and the number of published papers, smaller collections exist and have been used in functional studies such as a gamma-rays induced mutations (Sagan et al., 1995) and activation-tagging population (Porceddu et al., 2008).

### EMS population

An EMS population was generated by treating seeds using EMS, a chemical mutagen, inducing point mutations throughout the genome by C/G to A/T substitutions. EMS mutagenesis is very popular to generate mutant populations because of its ability in introducing high-density mutations. This population in *M. truncatula* genotype A17 has been extensively used in both forward and reverse genetic screens. It comprises almost 9000 M2 plants derived from 4500 M2 plants obtained from 500 M1 (not using single seed descent) and from 4350 M2 derived from 4350 M1 (using single seed descent; Le Signor et al., 2009). For reverse genetic screening, the population is screened using a Target Induced Local Lesion IN Genomes (TILLING) approach. This technique permits to localize point mutations in pooled genomic DNA sequences from various mutagenized plants. It requires the availability of genomic sequences and relies on a specific digestion enzyme (e.g., Cell), which is able to cleave heteroduplexes formed by the association of wild-type and mutated PCR products at the site of mismatch (Till et al., 2004). Two websites are associated to this population: the REVGENUK web-server (http://revgenuk.jic.ac.uk/order.htm) to request a reverse screen of your candidate gene and the phenotypic database (http://www.inra.fr/legumbase) to facilitate the identification of mutant line phenotypes and to request specific mutated lines.

### Fast neutron bombardment

Fast neutron bombardment (FNB) is another typical approach to mutate plant genomes. Unlike EMS, it results in DNA deletions ranging from a few bases to more than 30 Kb, and possible chromosomal rearrangements. This technique generates a high proportion of knockout mutations (i.e., KO mutations) but low mutation densities (Tadege et al., 2005). Thus, it requires large population sets to achieve saturation mutagenesis. For instance, *M. truncatula* FNB population consists of more than 80,000 M1 lines. Deletion-based TILLING (DeTILLING) has been established to identify the deletions within a large population (Rogers et al., 2009). The method adopts a three-dimensional pooling strategy together with PCR-based screening to enhance the efficiency of mutant recovery. All the information related to request a screen or to access to the phenotypes of mutated lines is available at the FNB dedicated webserver (http://bioinfo4.noble.org/mutant/).

### Insertional mutagenesis

Insertional mutagenesis is one of the most powerful approaches to obtain null mutants (i.e., KO) as demonstrated by the success of Arabidopsis T-DNA populations. In *M. truncatula*, the well-studied tobacco retro-transposon *Tnt1* was introduced into R108 genome, another genotype of *M. truncatula*. A study of its transposition indicated that *Tnt1* transposes actively during tissue culture (4–50 transpositions per genome) and that insertions are stable (D'Erfurth et al., 2003). Furthermore, *Tnt1* inserts were proven to preferentially target exons making a perfect tool to preferentially knocking out genes (D'Erfurth et al., 2003). Because of these characteristics, saturation mutagenesis of the *Tnt1* population requires a relatively small population. To date, the *M. truncatula* population comprises ~22,000 mutant lines and is near saturation with insertions in around 90% of all genes (Cheng et al., 2014). *Tnt1* mutant population has been used in forward genetic screens using inverse-PCR or thermal asymmetric interlaced-PCR (TAIL-PCR) to recover the flanking sequences of the insertion (Benlloch et al., 2006; Cheng et al., 2014). The use of this population in forward genetic screens was recently facilitated by the development of an algorithm, ITIS, which retrieves *Tnt1* insertion sites from mutant line genomes using low coverage genome sequencing data (Jiang et al., 2015). Meanwhile reverse genetic screening has been developed, including PCR-based DNA pool screening for candidate genes, and more recently using a BLAST database containing flanking sequence tags (FST; i.e., flanking regions of the *Tnt1* insertions) (Cheng et al., 2014). All the information regarding the *Tnt1* population such as request of mutated lines, description of mutant line phenotypes and a BLAST-able database of FSTs are available at http://medicago-mutant.noble.org/.

Besides the mutant populations described previously, other tools have shown their efficiency to functionally characterize multiple genes such as RNA interference approaches (RNAi) or the use of *MERE1* (*Medicago RetroElement1*), a low-copy retro-element naturally present in *M. truncatula* that showed transposition events preferentially in genic regions occurring during tissue culture (Rakocevic et al., 2009). Other strategies have emerged for functional characterization of genes and are under construction, such as the activation tag population, which consists of the introduction of a T-DNA together with the cauliflower mosaic virus (CaMV) 35S promoter into genome with the potential to generate gain-of-function phenotypes.

In this review, we will provide a non-exhaustive list of *M. truncatula* genes, which have been functionally characterized using one of the previously described mutant populations. To demonstrate that *M. truncatula* is a valuable model legume in broad research fields of plant biology, we decided to divide the functional studies into three important biological areas based on the number of published studies: (i) Rhizobia-legume symbiosis, (ii) seed biology, and (iii) abiotic stress biology.

## Functional genetics of rhizobia-legume symbiosis

### Lateral root and nodule development in *Medicago truncatula*

Plant root system is crucial for anchorage to the soil and acquisition of nutrients. In dicotyledonous plants, taproot architecture is mainly composed of an embryonically derived primary root and post-embryonically generated lateral roots. Both root nodules (an organ formed after the invasion by diazotrophs, see below for details) and lateral roots (LR) originate by dedifferentiation of a few cells in the pericycle and cortex of main root (Herrbach et al., 2014). LR development is influenced by symbiotic bacterial inoculation and nodule formation in legumes. Several plant and bacterial mutants have demonstrated abnormal nodule organogenesis featuring a central vascular bundle, like lateral roots (Guan et al., 2013). Together, these observations suggest that evolution of nodule development involved extensive recruitment and reengagement of pre-existing LR developmental pathways. Extensive functional genetic studies have been carried out to understand root nodule development in *Medicago* (Table 1).

**Table 1:** **List of nodule development related genes that have been functionally characterized in ***M. truncatula*****.

	**Gene**	**Gene full name**	**Mutant population**	**Proposed gene function/description**	**References**
Nod factor signaling and epidermal infection	*NFP*	*NOD FACTOR PERCEPTION*	EMS, RNAi	A LysM domain receptor kinase, putative NOD factor receptor	Madsen et al., 2003; Arrighi et al., 2006
	*DMI1*	*DOES NOT MAKE INFECTION 1*	EMS; Tnt1	Membrane depolarizing pump, needed for the generation of the self-sustaining Ca2+ spiking	Ané et al., 2004; Peiter et al., 2007
	*DMI2*	*DOES NOT MAKE INFECTION 2*	EMS; γ-rays; Tnt1	A LRR receptor kinase, required for bacterial recognition and endocytosis	Endre et al., 2002
	*HCL/LYK3*	*HAIR CURLING/LysM DOMAIN RECEPTOR KINASE 3*	EMS; RNAi, Tnt1	A LysM domain receptor kinase, putative NOD factor receptor	Limpens et al., 2003; Smit et al., 2007
	*SYMREM1*	*SYMBIOTIC REMORIN 1*	RNAi; Tnt1	Remorin protein, probable role in lipid micro-domain formation required for IT formation	Lefebvre et al., 2010
	*FLOT2*	*FLOTILLIN2*	RNAi	Flotillins are required for infection by nitrogen-fixing bacteria and probable role in membrane shaping	Haney and Long, 2010
	*FLOT4*	*FLOTILLIN4*	RNAi	Flotillins are required for infection by nitrogen-fixing bacteria and probable role in membrane shaping	Haney and Long, 2010
	*VPY*	*VAPYRIN*	FNB,Tnt1	Major sperm protein domain and a multiple of ankyrin repeats containing protein required for IT formation	Murray et al., 2010
	*CBS1*	*Cystathionine-β-Synthase-like1*	Tnt1	Cystathionine-β-Synthase and DUF21 domain containing protein, probable role in IT wall formation	Sinharoy et al., 2016
	*PUB1*	*M. truncatula Plant U-box protein 1*	RNAi	U-box containing a E3-ubiquitin ligase regulating rhizobial infection through protein degradation	Mbengue et al., 2010
	*MCA8*	*M. truncatula calcium ATPase 8*	RNAi	sarco/endoplasmic reticulum calcium ATPase, required for self-sustaining Ca2+ spiking	Capoen et al., 2011
	*IPD3*	*INTERACTING PROTEIN OF DMI3*	Tnt1	Transcription factor activate downstream gene expression	Horváth et al., 2011
	*NIN*	*NODULE INCEPTION*	FNB,Tnt1	RWP-RK domain containing transcription factor activate downstream gene expression	Marsh et al., 2007
	*HAP2-1/NF-YA1*	*CCAAT box binding transcription factor*	EMS	Nuclear transcription factor Y subunit alpha transcription factor activating required for Medicago nodule meristem development	Combier et al., 2006
	*NSP1*	*NODULATION SIGNALING PATHWAY 1*	EMS; Tnt1	GRAS domain containing transcription factor activating downstream gene expression	Smit et al., 2005
	*NSP2*	*NODULATION SIGNALING PATHWAY 2*	EMS; Tnt1	GRAS domain containing transcription factor activating downstream gene expression	Kaló et al., 2005
	*ERN1*	*ERF REQUIRED FOR NODULATION1*	FNB; EMS; Tnt1	AP2-ERF domain containing transcription factor required at several stages of nodule development	Middleton et al., 2007
	*ERN2*	*ERF REQUIRED FOR NODULATION 2*		AP2-ERF domain containing transcription factor required for infection thread development	Cerri et al., 2016
	*RPG*	*RHIZOBIUM-DIRECTED POLAR GROWTH*	EMS	Nuclear localized coiled-coil protein	Arrighi et al., 2008
	*RIT/NAP1*	*REQUIRED FOR INFECTION THREAD*	FNB/T-DNA	SCAR/WAVE complex, required for the actin polymerization through the activation of ARP2/3, played important role during IT propagation	Miyahara et al., 2010
	*LIN*	*LUMPY INFECTIONS*	Ac, EMS, T-DNA	U-Box, E3 ubiquitin ligase and WD40 repeat domains required for It propagation	Kiss et al., 2009
Nodule Organogenesis	*RR9*	*RESPONSE REGULATOR 9*	RNAi	Response regulator, working downstream of cytokinin signaling and controls nodule primordium development	Op den Camp et al., 2011
	*CRE1*	*CYTOKININ RESPONSE 1*	RNAi	Cytokinin receptor a histidine kinase promotes nodule primordium development	Gonzalez-Rizzo et al., 2006
	*DNF1*	*DEFECTIVE IN NITROGEN FIXATION 1*	FNB	Subunit of the signal peptidase complex, regulating protein trafficking toward symbiosome	Wang et al., 2010
	*DNF2*	*DEFECTIVE IN NITROGEN FIXATION 2*	FNB	Putative non-canonical phosphatidylinositol phospholipase C-like protein required to reduce defense response	Bourcy et al., 2013
	*DNF3*	*DEFECTIVE IN NITROGEN FIXATION 3*	FNB	Not known	Starker et al., 2006
	*DNF4*	*DEFECTIVE IN NITROGEN FIXATION 4*	FNB	NCR211 required for symbiosome maintenance	Kim et al., 2015
	*DNF5*	*DEFECTIVE IN NITROGEN FIXATION 5*	FNB, EMS	Not known	Starker et al., 2006; Domonkos et al., 2013
	*DNF6*	*DEFECTIVE IN NITROGEN FIXATION 6*	FNB	Not known	Starker et al., 2006
	*DNF7*	*DEFECTIVE IN NITROGEN FIXATION 7*	FNB	NCR169 required for symbiosome maintenance	Horváth et al., 2015
	*DNF8*	*DEFECTIVE IN NITROGEN FIXATION 8*	EMS	Not known	Domonkos et al., 2013
	*5L/11S*	*Fix- mutant*	EMS	Required for the maintenance of bacterial infection	Domonkos et al., 2013
	*7Y*	*Fix- mutant*	EMS	Required for the maintenance of bacterial infection	Domonkos et al., 2013
	*13U*	*Fix- mutant*	EMS	Required for the maintenance of bacterial infection	Domonkos et al., 2013
	*SYMCRK*	*Symbiosis cysteine-rich receptor-like kinase*	Tnt1	Cysteine-rich nonRD receptor-like kinase repress defense-like reactions in nodules	Berrabah et al., 2014
	*RSD*	*REGULATOR OF SYMBIOSOME DIFFERENTIATION*	Tnt1	C_2_H_2_ transcription factor regulating symbiosome development	Sinharoy et al., 2013
	*NAD1*	*Nodules with activated defense 1*	Tnt1	Nodules with activated defense 1 repress defense-like reactions in nodules	Wang et al., 2016
	*EFD*	*Ethylene response factor required for nodule differentiation*	FNB	Required for the formation of functional nitrogen-fixing nodules and controlling nodule numbers	Vernie et al., 2008
Local control of nodule number	*SKL1*	*SICKLE*	EMS; Tnt1	NRAMP-like integral membrane protein located at the endoplasmic reticulum. Controls nodule numbers	Penmetsa et al., 2008
	*CEPs*	*C-TERMINALLY ENCODED PEPTIDEs*	RNAi and overexpression	signaling peptide family, positively regulate nodule number and negatively regulate lateral root development	Imin et al., 2013
Autoregulation of Nodulation	*SUNN*	*Super numerary nodules*	EMS; γ-rays; Tnt1	Leucine-rich repeat receptor kinase like CLAVATA1 (CLV1) control nodule number	Schnabel et al., 2005
	*MtCLE12*	*CLV3/ESR-related 12*	Ectopically expressed in transgenic roots	Controls nodule number in SUNN dependent manner	Mortier et al., 2010
	*MtCLE13*	*CLV3/ESR-related 13*	ectopically expressed in transgenic roots	Controls nodule number in SUNN dependent manner	Mortier et al., 2010
	*RDN1*	*Root Determined Nodulation 1*	FNB	Most probably RDN1 glycosylates CLE peptide(s) and controls nodule numbers	Kassaw et al., 2015
	*LSS*	*Like sunn supernodulator*	spontaneously occurring super nodulation mutant from the Jemalong cultivar	*Lss* is probably a cis-acting factor that inhibits the expression of SUNN	Schnabel et al., 2010
	*CRA2*	*compact root architecture 2*	Tnt1	Leucine-Rich Repeat Receptor-Like Kinase, negatively regulates lateral root development and positively regulates nodulation	Huault et al., 2014
	*MtCLV2*	*CLAVATA2*	Interaction study	Leucine-rich repeat receptor-like kinase, probably negatively regulate nodule number	Crook et al., 2016
	*MtCRN*	*CORYNE*	Tnt1	Leucine-rich repeat receptor-like kinase, negatively regulate nodule number	Crook et al., 2016

### Root nodule development in *Medicago truncatula*

Development of nodules is initiated by an exchange of chemical signals between plant cells and soil bacteria called rhizobia (Oldroyd, 2013). In most of the legumes including *Medicago*, rhizobia produce and release lipochitooligosaccharides called Nod factors (NF) in response to flavonoids secreted by the host. NF are perceived by the root hair cells. Perception of NF leads to the induction of a calcium (Ca^+2^) influx and is followed by Ca^+2^ oscillations around the nucleus of the root hair cell. All these molecular changes lead to asymmetric growth (curling) of the root hair cells and division of cortical cells (Limpens et al., 2003; Radutoiu et al., 2003). Rhizobia enter and traverse epidermal and underlying cortical cells via a plant cell wall and plasma membrane invagination called the infection thread (ITs). Bacteria are eventually released into the cytoplasm of nodule primordial cells by endocytosis. Rhizobial endocytosis results in bacteria surrounded by a plant membrane, called the symbiosome membrane (SM), creating a novel organelle called symbiosome (Goodchild and Bergersen, 1966; Robertson and Lyttleton, 1984; Roth and Stacey, 1989). Finally, rhizobia (now called the bacteroids) undergo a process of differentiation that involves induction of nitrogen fixation genes and repression of ammonium assimilation genes, turning symbiosomes into an ammonium-exporting organelle (Udvardi and Day, 1997; Udvardi and Poole, 2013).

Nodules in *M. truncatula* always retain the meristem and keep on growing (indeterminate type). A mature nodule is organized into five well-defined developmental zones: Zone I (meristem), Zone II (invasion zone), Zone II-III (interzone), Zone III (nitrogen fixation zone), and Zone IV (senescent zone, only present in older nodules). Bacteroid genomes in *M. truncatula* undergo several rounds of replication without any cell division (endoreduplication), which leads to polyploidy of the bacteroids. Endoreduplication leads to the formation of elongated or Y-shaped bacteroids. Both bacterial cell division and elongated or Y-shaped bacteroid formation take place in the invasion zone (Oldroyd, 2013). Several parallel approaches have been undertaken to understand nodule development in *M. truncatula*. Two main methods have been forward genetic approach and transcriptomic analyses followed by reverse genetic approach. Extensive forward genetic and biochemical studies have been performed to understand the molecular mechanism behind nodule development. In the last decade, a rapid advancement of functional genomic technology helped us better understand the process of nodule development.

**Figure 1:**
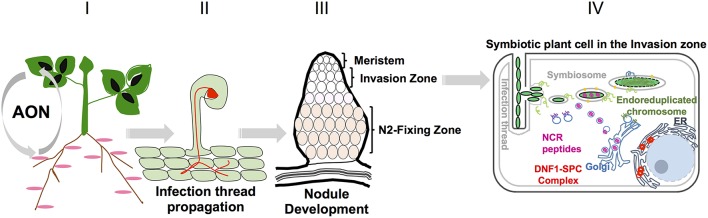
**Different nodule developmental stages in ***Medicago***: I Nodule number is controlled systemically by long distance signaling from root to shoot and back again to root, called autoregulation of nodulation (AON)**. II Rhizobia enter the root epidermis through the formation of infection threads. III A fully mature *Medicago* nodule with different nodule zones. Bacteroid maturation takes place in the invasion zone and nitrogen fixation takes place in nitrogen fixation zone. IV Schematic representation of one infected cell in the invasion zone. Bacterial endocytosis, differentiation to functional bacteroids takes place in this specific zone.

A schematic description of nodule formation, nodule zonation and symbiotic plant cell of the invasion zone is provided in Figure 1.

### NOD factor signaling and epidermal infection

#### Signaling at the plasma membrane of the root hair cell

A systematic mutagenesis approach has been undertaken in *M. truncatula* Jemalong A17 using EMS (Penmetsa and Cook, 2000) to unravel the major players behind nodule development. Mutation in LysM domain containing receptor-like kinase NOD FACTOR PERCEPTION (NFP; Amor et al., 2003) eliminates any response in the root hair cells in presence of rhizobia/NF. Two other genes involved in nuclear-associated Ca^+2^ spiking response are *DOES NOT MAKE INFECTION* (*DMI*) genes *DMI1* and *DMI2* (Endre et al., 2002; Ané et al., 2004). *dmi1* and *dmi2* respond to NFs by causing swelling of the root hair in the absence of Ca^+2^ spiking (Ané et al., 2002). *dmi2* encodes a leucine rich receptor (LRR) domain containing receptor-like kinase and acts in parallel with NFP. Another membrane-localized LysM domain containing receptor-like kinase has been implicated in NF recognition. This gene is named *HAIR CURLING* (*HCL*), and it encodes a LysM DOMAIN RECEPTOR KINASE 3 (LYK3; Smit et al., 2007). Excessive root hair curling and cortical cell division occurs in the *hcl/lyk3* mutant, but rhizobia are not entrapped into the root curls.

In a reverse genetic approach, *SYMBIOTIC REMORIN 1* (*MtSYMREM1*) has been identified. The above-mentioned three membrane-bound kinases (DMI2, NFP, and LYK3) interact with MtSYMREM1. The mutant of MtSYMREM1 was isolated from *Tnt1* mutant population, and its phenotype indicated its involvement during bacterial endocytosis rather than IT formation (Lefebvre et al., 2010). Another gene family that has been implicated in *M. truncatula* IT development by reverse genetic studies is “*FLOTILLIN*.” Flotillins are peripheral membrane proteins known to be required for endocytosis and membrane shaping. Two flotillin genes *FLOT2* and *FLOT4* are induced downstream of the NF signaling pathway. Silencing of *FLOT2* and *FLOT4* expression revealed a non-redundant requirement for both genes in IT initiation and nodule development (Haney and Long, 2010). The *VAPYRIN* (*VPY*) gene encodes a protein with a major sperm protein domain and a series of ankyrin repeats. Ankyrin repeats are typically found in proteins involved in membrane trafficking and biogenesis. The *vpy* mutant showed abnormal IT formation and fewer underdeveloped nodules (Murray et al., 2010). Mutation in *Cystathionine-β-Synthase-like1* (*CBS1)* causes a defect in the infection thread propagation. *cbs1* plants have more micro-colonies but less propagating infection threads. CBS1 encodes for a putative membrane-localized domain of unknown function (DUF21) and a cystathionine-β-synthase domain, and is has been speculated that CBS1 participate in infection thread cell wall development (Sinharoy et al., 2016).

In summary, the three receptor-like kinases (DMI2, NFP, and LYK3) and FLOT4 probably localize at the tip of the root hair cells. They change their localization and organize themselves into a microdomain after the application of NFs (Haney et al., 2011). These, in turn, activate a nuclear Ca^+2^ spiking that acts as a secondary messenger to activate gene expression in the nucleus. Interaction of LYK3 with an *M. truncatula* Plant U-box protein 1 (PUB1, a E3-ubiquitin ligase) suggests that the initial signaling involves protein degradation (Mbengue et al., 2010). Recently, it has been shown that PUB1 interacts and is phosphorylated by DMI2, which supports the findings that the concerted action of the receptor kinases controls rhizobial entry (Vernié et al., 2016). The actual function of VAPYRIN and CBS1 in this context is not clear.

#### Calcium spiking in the nucleus

DMI1 is an inner-membrane-localized channel protein. DMI1 localizes to the ER membrane, and the ER is one of the major calcium storages in the cell (Ané et al., 2004). A Ca^2+^ pump (MCA8, sarco/endoplasmic reticulum calcium ATPase), three cyclic nucleotide-gated channels (CNGC15a, CNGC15b, CNGC15c) and potassium-permeable channel (DMI1) are needed for the generation of the self-sustaining Ca^2+^ spiking. The three cyclic nucleotide-gated channels form a complex with the DMI1 in nuclear envelope, which modulates nuclear Ca(2+) release (Charpentier et al., 2016). All the three CNGC15a-c and MCA8 has been identified by a reverse genetic approach (Capoen et al., 2011).

#### Gene expression

A Ca^2+^/calmodulin dependent protein kinase, named DMI3, acts downstream to the symbiotic Ca^2+^ spiking. DMI3 has an exceptional ability to bind free Ca^2+^ both directly using EF-hand domains and indirectly through a calmodulin (CaM) binding domain. It has been hypothesized that DMI3 decodes the calcium spiking signal that leads to nodule development. Indeed, the *dmi3* mutant has normal Ca^2+^ spiking responses but does not form any nodules. Several transcription factors acting downstream of DMI3 have been identified (Oldroyd, 2013). Among them, *INTERACTING PROTEIN OF DMI3* (*IPD3*) represents a unique class of transcription factor family. DMI3 phosphorylates IPD3, leading to the activation of the latter, and eventually activation of its downstream targets like *NODULE INCEPTION* (*NIN*) and a CCAAT-box binding transcription factor (*HAP2-1*; Oldroyd, 2013; Singh et al., 2014).

Several other transcription factors have been implicated in nodule development. Plants showing mutation(s) in these genes either develop small bump-like nodules without bacterial colonization or no nodules. *NODULATION SIGNALING PATHWAY* genes (*NSP1* and *NSP2*) are plant specific GRAS transcription factor/regulators. *nsp1* and *nsp2* mutants show root hair deformation but the induction of cortical cells in response to NF is blocked (Oldroyd and Long, 2003; Kaló et al., 2005; Smit et al., 2005). Three more TFs have been implicated in this signaling process. Among them, *ERF REQUIRED FOR NODULATION* (*ERN1*) contains a highly conserved AP2-DNA binding domain. *ern1* initiate the development of ITs but still form small bumps (Middleton et al., 2007). *Medicago ERN1* has a close homolog, *ERN2*. ERN1/ERN2 act in concert in the root epidermis and the *ern1/ern2* double mutant displays a severe phenotype where the initiation of infection is completely abolished (Cerri et al., 2016). MtNF-YA1/HAP2-1 is a *Medicago* CCAAT box-binding TF. The *nf-ya1* was isolated from an EMS mutant population. This gene is required to initiate IT formation and to maintain the persistent meristem activity in mature nodules (Laporte et al., 2014). Probably, the most vital transcription factor that controls epidermal infection, cortical cell division and nodule number is *NIN. nin* mutants undergo excessive root hair curling in response to inoculation by *Sinorhizobium*, but are impaired in infection and do not show any cortical cell division (Marsh et al., 2007; Soyano et al., 2014; Yoro et al., 2014). *RHIZOBIUM-DIRECTED POLAR GROWTH* (*RPG)* gene of *M. truncatula* is also worth mentioning. Nitrogen-fixing nodules are rarely formed in *rpg* mutants. ITs are abnormally thick and progress slowly; moreover, root hair curling is abnormal. This gene encodes for a yet uncharacterized putative long coiled-coil protein. This protein has a nuclear localization signal (NLS) and was found to actually localize to the nucleus in *Nicotiana* cells (Arrighi et al., 2008). The actual role of this gene is not clear to date.

### Events that take place in root hair cells following bacterial inoculation

The first thing that is apparent from the complexity of IT development is the need of cytoskeleton rearrangement. In a forward genetic approach, *M. truncatula REQUIRED FOR INFECTION THREAD* (*rit/nap1)* mutant has been isolated, which encodes for a component of the SCAR/WAVE (suppressor of cAMP receptor/WASP-family verprolin homologous protein) complex. This complex regulates actin polymerization through the activation of ARP2/3, suggesting that actin cytoskeleton rearrangement is crucial for IT propagation (Miyahara et al., 2010). *LUMPY INFECTIONS* (*LIN*) is another gene required for the growth of ITs in the root hair cells. It encodes for a protein with *U*-Box, E3 ubiquitin ligase and WD40 repeat domains. This also suggests that protein degradation is a crucial mechanism downstream of NF signaling in the root hair cells (Kiss et al., 2009).

Although the last two decades have brought significant increase in our knowledge of the early infection process, a lot of unanswered questions still remain. A transcriptional profiling of *M. truncatula* root hairs prior to and during the initial stages of rhizobial infection has been undertaken (Breakspear et al., 2014). A Systems biology approach of this single cell model revealed (a) activation of plant cell cycle, (b) involvement of several hormone related pathways like auxin, gibberellin, strigolactone, brassinosteroid, jasmonic acid and salicylic acid, and (c) expression of infection specific flavonoid synthesis genes and bacterial NF degradation genes. From this study, it appears that at the onset of infection, the plant cell cycle is reactivated, and along with that, hormone and flavonoid biosynthesis is necessary for rhizobial infection. It also suggests that both positive and negative feedback loops control levels of NF during rhizobial infection. Further genetic studies are needed to find out unique and redundant pathways that are required during bacterial infection (Breakspear et al., 2014).

### Nodule organogenesis

#### Infection and nodule development are uncoupled

The first genetic evidence that highlighted the fact that bacterial infection and nodule development are two independent phenomena came from *Lotus japonicus*. In *Lotus, snf1* and *snf2* mutants initiated nodule development independently of any rhizobial inoculation (Tirichine et al., 2006, 2007). *Lotus snf1* encodes for CCaMK, which is orthologous to *M. truncatula* DMI3. Deregulation of the kinase activity of DMI3 leads to auto-nodule development in *M. truncatula* as well (Gleason et al., 2006). DMI3 has a dual sensing module, which is able to perceive the Ca^2+^ spiking and to promote the downstream NF signaling pathway. DMI3 controls bacterial infection in the epidermis and promotes nodule organogenesis. If the tuning of the kinase activity is hampered, it would lead to activation of the NF signaling pathway (Miller et al., 2013). The second report of the cortical cell division in absence of rhizobia came from the overexpression of *RESPONSE REGULATOR 9* (*RR9*) in *M. truncatula*. RR9 is a response regulator that acts downstream of cytokinin signaling. Overexpression of *RR9* leads to cortical cell division and generation of a bulge like structure in roots (Op den Camp et al., 2011). Involvement of cytokinin in spontaneous nodule development was reported earlier in *Lotus*. *snf2* is a *Lotus* cytokinin receptor and have a histidine kinase domain. A point mutation in the cytokinin perceiving domain leads to auto-activation of its kinase activity, causing spontaneous nodule development in *Lotus* (Tirichine et al., 2007). The orthologous gene of *Lotus snf2* has been identified in *M. truncatula* and named *CYTOKININ RESPONSE 1* (*CRE1*; Gonzalez-Rizzo et al., 2006). *cre1* mutants show an early inhibition of cortical cell divisions during nodule initiation (Gonzalez-Rizzo et al., 2006). The third report that shows spontaneous nodulation in *M. truncatula* came from the overexpression of the intracellular kinase-domain DMI2. Deregulation of DMI2 activity leads to hyper-activation of the nodule organogenesis program (Saha et al., 2014).

The overexpression of RWP-RK transcription factor (i.e., *NIN*) in *Lotus* is sufficient to induce spontaneous nodule-like structures (Soyano et al., 2013). Further, specific overexpression of *NIN* separately in epidermis (using an epidermal specific promoter) and in cortex (using a cortical specific promoter) promotes spontaneous nodules in *Medicago*. Additionally, NIN directly binds to the promoter of *CRE1* gene and induces cytokinin signaling. These results suggest that cytokinin is the best described downstream factor involved in spontaneous nodule organogenesis (Vernié et al., 2015).

In summary, nodule organogenesis is spatio-temporally separated into two sophistically regulated events, an epidermal bacterial infection and cortical cell division. Disruption of this regulation leads to spontaneous nodule development in both *Medicago* and *Lotus*. It appears that phosphorylation of the downstream targets induces activation of series of transcription factors, including *NIN*, which directly activates the expression of a cytokinin receptor (*CRE1*) in the cortex followed by the downstream cytokinin signaling inducing nodule development.

### Endocytosis of bacteria and symbiosome formation

#### NF signaling in bacterial endocytosis

In a wild type context, nodule development, and bacterial endocytosis are tightly regulated. ITs start to penetrate the dividing cortical cells after ~33–35 h of bacterial inoculation. Eventually, bacterial endocytosis takes place (Xiao et al., 2014). NF signaling module that operates in the epidermis during bacterial entrapment is also functional during the bacterial release process in the host cell cytoplasm. Specific knock-down of the *NFP* gene inside the nodule highlights its requirement for the release of the bacteria from the ITs. Knocking-down *DMI2* specifically inside the nodule prevents bacterial endocytosis. Rice CCaMK can restore the epidermal block in the *dmi3* mutant, but cannot rescue bacterial endocytosis (Godfroy et al., 2006). In the *ipd3-1*, bacteria are entrapped inside the ITs and nearly never released into the nodule primordium (Horváth et al., 2011). Thus, NFP-DMI2-DMI3 and IPD3 act in nodule epidermis and cortex during the plant-bacteria recognition phase.

#### Symbiosome formation

Rhizobia are released inside the host cytoplasm and form symbiosomes after about 80 h of inoculation (Xiao et al., 2014). Symbiosomes undergo several rounds of division followed by endoreduplication. Endoreduplication of the bacteroid genome in *M. truncatula* leads to elongated or Y-shaped bacteroids (called terminally differentiated). Simultaneously, host cortical cells undergo several rounds of endoreduplication (Oldroyd, 2013). A transcriptomic approach has been undertaken to understand the connection between the cellular differentiation and the transcriptional activation during nodule development (Maunoury et al., 2010). This study unraveled two waves of transcriptional activation. The first wave starts with the repression of plant defense-related genes and simultaneous activation of cell cycle and protein synthesis genes. The second wave is marked by the induction of secretory pathways along with a large number of small peptides, secretory and transmembrane proteins.

Several forward genetic studies also support the above-mentioned hypothesis of transcription reprogramming. Mutant lines with normal bacterial infection in the epidermis but impaired in nitrogen fixation are called DEFECTIVE IN NITROGEN FIXATION (*dnf* mutants). Eleven mutants belonging to the *dnf* category have been described, to date, namely *dnf1* to *dnf8*, 5L/11S, 7Y, and 13U (Starker et al., 2006; Domonkos et al., 2013). Among them, the genes responsible for *dnf1, dnf2* phenotypes have been identified. Bacteroids in *dnf1* remain small like their free-living counterparts, unlike the Y-shaped bacteroids in wild type. *DNF1* mutated gene encodes a subunit of the signal peptidase complex without the signal peptide of secretory proteins. *DNF1* is involved in protein trafficking to symbiosomes (Wang et al., 2010). Around 600 small peptide genes (NCR peptides) are responsible for the elongation of bacteroids and are present in *M. truncatula* but not in *L. japonicus* (Mergaert et al., 2003). Two NCR peptides, NCR169 and NCR211 are required for symbiotic nitrogen fixation in *M. truncatula* nodules (Horváth et al., 2015; Kim et al., 2015). At least, few NCR peptides are transported inside the symbiosome and DNF1 is required for their transport (Van de Velde et al., 2010). *dnf2* nodules have a narrow zone with infected cells. Nonetheless, improper differentiation of bacteroids results in rapidly prematurely senescing nodules. *DNF2* is a putative non-canonical phosphatidylinositol phospholipase C-like protein, which lacks its Y domain. DNF2 has N-terminal signal peptide, indicating its probable entry to the secretory pathway. It is proposed that DNF2 is required for the repression of defense response, but the actual role of this protein in nodule development remains elusive (Bourcy et al., 2013).

Two more crucial genes for nodule development have been identified in a reverse genetic approach [based on *Mt Gene Expression Atlas* Benedito et al., 2008]. One of them is *symCRK*. *symcrk* mutant plants develops non-functional nodules. *symcrk* nodules show defense-like reactions and early senescence. The predicted protein encodes for a membrane-bound kinase, which contains two extracellular cysteine-rich domains. The exact role of this protein in nodule development has not been understood, but it is predicted that symCRK may be involved in suppression of host defense response (Berrabah et al., 2014). The second one is a transcription factor, *REGULATOR OF SYMBIOSOME DIFFERENTIATION* (*RSD*). *RSD* encodes for a Cysteine-2/Histidine-2 (C_2_H_2_) zinc-finger family of plant TFs. In *rsd-1* mutant nodules, bacteroids fail to differentiate normally. The endoreduplication and the viability of the bacteroids are also compromised. MtRSD is a transcriptional regulator of plant secretory pathway genes *VAMP721a* and thereby controls symbiosome development (Sinharoy et al., 2013). RSD is also implicated in the repression of plant defenses in nodules (Berrabah et al., 2015) and predicted to work similarly as symCRK in defense suppression. Recently, another *M. truncatula* mutant has been described via reverse genetics, called *NODULE WITH ACTIVATED DEFENSE 1* (*NAD1*). *nad1* nodules shows very early defense response. The rhizobia, along with their symbiotic plant cells, become necrotic immediately after the bacteria are released from infection threads into the nodule symbiotic cells. NAD1 encodes a small and uncharacterized protein with two predicted transmembrane helices and is localized at the endoplasmic reticulum (Wang et al., 2016). Presence of high load of NCR peptides and their involvement in nodule development confer to *M. truncatula* a unique situation. The coherent action of DNF2, RSD, symCRK, NAD1, NCR peptides, and most probably several hundred uncharacterized genes, act together to repress the defense response and enhance efficient symbiotic nitrogen fixation after bacterial endocytosis. Detailed studies should be performed in order to unravel the molecular mechanism. Another transcription factor, *EFD* (*E**thylene response* *f**actor required for nodule* *d**ifferentiation*) controls nodule number by negatively regulating nodule development. This gene has been proposed to be involved in the bacteroid differentiation processes by activating MtRR4, a type-A cytokinin primary response regulator (Vernie et al., 2008).

#### Autoregulation of nodulation

Functional nodule development is a highly energy consuming process. The host plant spends up to 25% of a legume's net photosynthate for root nodule development (Oono and Denison, 2010). To efficiently manage the distribution of energy, plants developed a mechanism to control nodule number by terminating nodule development. This mechanism is called autoregulation of nodulation (AON; Kosslak and Bohlool, 1984). AON involves through long distance signaling, from root to shoot and back again to root. AON was discovered by the classical split root inoculation experiments, where one part of the split root was inoculated inducing a systemic suppression of nodule development on the other part of the split root (Kassaw et al., 2015).

More recently, genetic studies have been valuable to dissect the AON pathway. *M. truncatula* the *sickle* mutant lines display a drastically increased underdeveloped number of nodules. *SICKLE* gene encodes an integral membrane protein comprising an N-terminal segment that shows similarity with a family of metal transporters and a unique C-terminal segment (CEND, the C-terminal end of EIN2) that does not contain a known and characterized motif (Alonso et al., 1999; Penmetsa et al., 2008). The EIN2 (ETHYLENE INSENSITIVE2) protein was previously identified in *Arabidopsis*. Studies from *Arabidopsis* and several other plants highlighted that EIN2 plays a central role in ethylene signaling. After, the first phase of infection, the competency for further root hair mediated infection is controlled locally by SICKLE/EIN2-dependent pathway. Transcriptional profiling of the *Mtein2/sickle* mutant highlighted a SICKLE/EIN2 dependent pathway negatively controls NF-mediated nodule organogenesis (Lauressergues et al., 2015). The AON pathway is shoot-controlled and work systemically instead of locally like SICKLE/EIN2. The first AON gene that was cloned is *SUPER NUMERARY NODULES* (*SUNN)*, encoding a leucine-rich repeat receptor kinase like CLAVATA1 (CLV1) of *Arabidopsis thaliana* (Schnabel et al., 2005). The *sunn* mutants display a super-nodulation phenotype (van Noorden et al., 2006). Another shoot controlled super-nodulation mutant was identified and called *like sunn supernodulator* (*lss*). Transcriptomics studies highlighted that the main cause behind the *lss* phenotype is reduced *SUNN* expression. The prevailing hypothesis suggests that *lss* is a cis-acting factor that inhibits the expression of *SUNN* (Schnabel et al., 2010).

As mentioned earlier, AON involves long distance signaling from root to shoot with feedback signal to root. The root-derived signal is perceived by *SUNN* receptor kinase (Leucine-Rich Repeat Receptor-Like Kinase [LRR-RLK]). In *Arabidopsis* CLV1 receptor perceives short secreted CLE peptides (CLAVATA3/Endosperm surrounding region-related) and controls several aspects of plant development. *Arabidopsis* CLV1 functions within a larger receptor complex. AtCLV1-AtCLV2 heterodimer perceives AtCLV3 peptide (Jeong et al., 1999). Again, AtCLV2 forms a stable heterodimer with a membrane bound pseudokinase, CORYNE (CRN). AtCLV2-AtCRN pathway is able to function independently of AtCLV1 (Bleckmann et al., 2010). Over-expression of two CLE peptides (MtCLE12/MtCLE13 and LjCLE-RS1/LjCLE-RS2) systemically reduces nodule number in a MtCLV1-like receptor kinase dependent manner (Okamoto et al., 2009; Mortier et al., 2010). *LjCLE-RS1* and *LjCLE-RS2* were shown as direct targets of LjNIN in *L. japonicus* (Soyano et al., 2014). Expression of *NIN* is significantly reduced as a systemic effect of a CLE peptide over-expression in Medicago. It is still not clear whether or not CLE peptide genes are direct target of NIN in Medicago (Mortier et al., 2010). In a recent study, it has been shown that MtSUNN form homomers, and heteromers (with both MtCRN and MtCLV2). Further, in Medicago the *crn* mutant, shoot controlled AON is destroyed and it forms increased number of nodules. Indicate, that the same trio that control Arabidopsis shoot development, recruited to controls nodule number in Medicago. Another gene that has been identified in *M. truncatula* is *ROOT DETERMINED NODULATION 1* (*RDN1*; Schnabel et al., 2011). *RDN1* belongs to the HPAT gene family of *Arabidopsis*. In *Arabidopsis*, HPAT genes are able to glycosylate some of the CLE peptide (Ogawa-Ohnishi et al., 2013).

Conversely, C-TERMINALLY ENCODED PEPTIDEs (CEPs) positively regulate nodule number and negatively regulate lateral root emergence locally (Imin et al., 2013). Again, compact root architecture (*cra2*) mutant have more lateral roots and reduced number of nodules. The CRA2 gene encodes another a LRR-RLK. Like CEPs, CRA2 negatively regulates lateral root formation and positively regulates symbiotic nodulation (Huault et al., 2014). In a recent study it has been shown that unlike in wild-type, application of MtCEP1 is unable to increase nodule number in *cra2* mutant (Mohd-Radzman et al., 2016). CRA2 is very closely related to *A. thaliana* XIP1 (XYLEM INTERMIXED IN PHLOEM1) receptor (Bryan et al., 2012) which specifically binds CEPs. Taking together the knowledge from *Medicago* and *Arabidopsis* it appears that MtCEPs can bind to MtCRA2 receptor and regulate nodule number. MtCEP1 treatment can increase nodule number in the *sunn* mutant but failed to increase nodule number in the *sickle* background, suggest there is an overlap between CEP medicated and EIN2 dependent nodule number controlling pathway (Crook et al., 2016).

Double mutant analysis using *sickle* and *sunn* mutants highlighted that *SICKLE* and *SUNN* participate in distinct genetic pathways (Penmetsa et al., 2003). Ectopic overexpression of the cytoplasmic kinase domain of DMI2 in *sickle* background increases the hyper nodulation phenotype, but it completely abolishes nodule development in *sunn* background. This confirms, SUNN-mediated systemic AON signaling pathway and SICKLE-mediated local nodule number control pathway acts through separate pathways (Saha and DasGupta, 2015). This result also suggests that DMI2 can be another determinant controlling nodule number.

To summarize, combined negative action of SICKLE and SUNN mediated pathways and positive action CRA2 and CEPs medicated induction of nodule development, tightly control nodule number. The AON mechanism starts very early during nodule development, probably MtNIN activates CLE peptides gene expression. CLE peptide repress *NIN* expression in a negative feedback loop. Most likely, SUNN receptor kinase act as a receptor of RDN1 glycosylate CLE peptide, CRA2 and CEPs medicated positive regulation of nodule number work through SICKLE and positively regulate nodule number.

## Functional genetics of seed development

**Figure 2:**
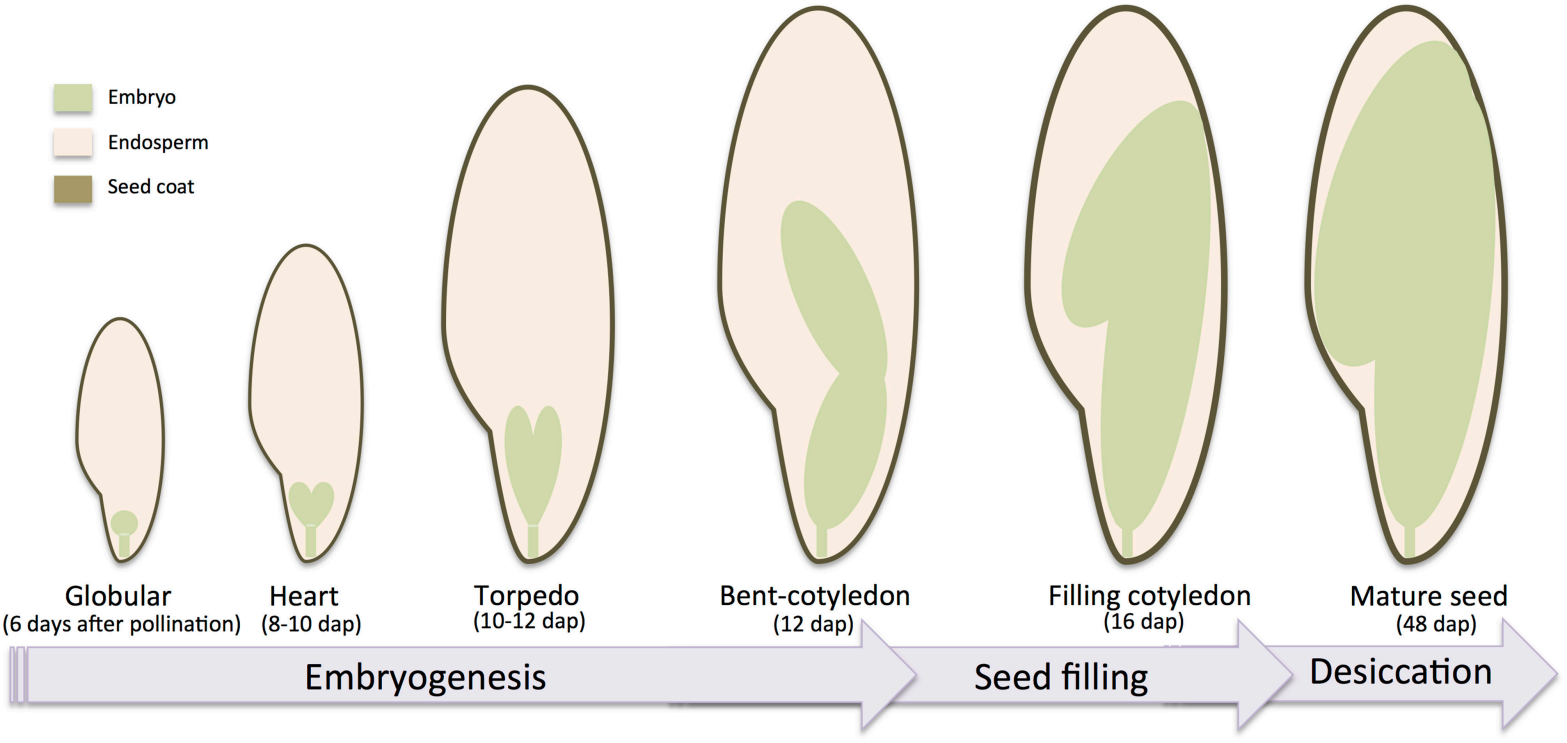
**Schematic development of ***Medicago truncatula*** seed**. Major seed tissues and developmental seed and embryo stages are indicated with corresponding days after pollination.

Given the economical and nutritional importance of legume seeds, seed development of *M. truncatula* has been intensively studied and various reports have been published regarding the accumulation of transcripts (Gallardo et al., 2007; Benedito et al., 2008; Verdier et al., 2008), proteins (Gallardo et al., 2003), lipids and sugars (Djemel et al., 2005), and metabolites (Verdier et al., 2013b) during the development of the seeds. Seed development is usually divided into three phases: the embryogenesis marked by the development of the embryo, the early maturation or seed filling marked by the accumulation of storage macromolecules in cotyledons, and the late maturation marked by the desiccation of the seed and its entry into dormancy. In *M. truncatula*, several reports (Gallardo et al., 2007; Verdier et al., 2008) described the timing of seed development with embryogenesis starting at the double fertilization until 12–13 days after pollination (dap), then the early maturation until around 20 dap, and finally the late maturation until the seed become mature at ~48 dap. The seed is also characterized according to its tissues: the embryo, the endosperm and the seed coat. The formation of these tissues is derived from the double fertilization (Goldberg et al., 1994). The integument of the ovule develops into seed coat, and the inside of the ovule progresses to embryo sac. Within the sac, the central cell (2n) fuses with one sperm cell (1n) to form the triploid endosperm. In the meanwhile, the egg cell (1n) together with another sperm cell (1n) will form the diploid zygote, which will go through the embryogenesis. A schematic description of the seed tissues and seed developmental stages with the timing of *M. truncatula* seed development is provided in Figure 2.

In this section, we classified the gene functional studies into three subfields related to their role during seed development: genes controlling embryogenesis, seed composition and desiccation tolerance. All the genes proposed in the following section are described in Table 2.

**Table 2:** **List of seed development related genes that have been functionally characterized in ***M. truncatula*****.

	**Gene**	**Gene full name**	**Mutant population**	**Proposed gene function/description**	**References**
Embryogenesis	*MtSERF1*	*Somatic Embryo Related Factor1*	RNA interference	Essential for somatic embryogenesis	Mantiri et al., 2008
	*MtWUS*	*Wuschel*	RNA interference	Essential for somatic embryogenesis	Chen et al., 2009
	*SBT1.1*	*Subtilase1*	TILLING population	Control of cell division within the embryo	D'Erfurth et al., 2012
	*MtDASH*	*Dof Acting in Seed embryogenesis and Hormone accumulation*	*Tnt*1-insertion mutant; EMS-TILLING population	Control of cell division within the embryo	Noguero et al., 2015
Seed composition	*UGT78G1*	*UDP-Glucosyl transferase 78G1*	*Tnt*1-insertion mutant	Accumulation of anthocyanins	Peel et al., 2009
	*MtLAP1*	*Legume Anthocyanin production 1*	Over-expression	Accumulation of anthocyanins	Peel et al., 2009
	*MATE1*	*Multidrug And Toxic compound Extrusion 1*	*Tnt*1-insertion mutant	Accumulation of PAs	Zhao and Dixon, 2009
	*MATE2*	*Multidrug And Toxic compound Extrusion 2*	*Tnt*1-insertion mutant	Anthocyanin transport	Zhao et al., 2011
	*MtSAP1*	*Stress-Associated protein 1*	RNA interference	Essential for proper storage seed protein accumulation	Gimeno-Gilles et al., 2011
	*MtPAR*	*ProAnthocyanidin Regulator*	*Tnt*1-insertion mutant	Accumulation of PAs	Verdier et al., 2012
	*UGT72L1*	*UDP-Glucosyl transferase 72L1*	*Tnt*1-insertion mutant	Accumulation of PAs	Pang et al., 2013
	*MtMYB5*	*MYB5*	*Tnt*1-insertion mutant	Accumulation of PAs	Liu et al., 2014
	*MtMYB14*	*MYB14*	*Tnt*1-insertion mutant	Accumulation of PAs	Liu et al., 2014
Desiccation tolerance	*MtSNF4b*	*Sucrose Non-Fermenting 4b*	RNA interference	Regulation of seed longevity	Rosnoblet et al., 2007
	*MtSGR*	*Stay Green*	*Tnt*1-insertion mutant	Seed desiccation tolerance	Zhou et al., 2011
	*MtABI3*	*Abscisic Acid Insensitive 3*	*Tnt*1-insertion mutant	Seed desiccation tolerance	Delahaie et al., 2013
	*MtABI5*	*Abscisic Acid Insensitive 5*	*Tnt*1-insertion mutant	Seed desiccation tolerance	Terrasson et al., 2013

### Embryogenesis

Embryogenesis is a very well-studied process in plants and many genes are necessary for the acquisition of the polarity and the morphogenesis. For instance, it has been shown in Arabidopsis that more than 400 genes are required for the proper development of the embryo (Muralla et al., 2011). In *M. truncatula*, the formation of the embryo starts after the double fertilization event and the embryo goes through the different developmental stages known as globular (around 6 dap), heart (around 8–10 dap), torpedo (around 10–11 dap), and reaches the bent cotyledon stage around 12 DAP marking the end of the embryogenesis (Verdier et al., 2013a; Noguero et al., 2015; Figure 2). Some of the essential genes required for *M. truncatula* embryo development have been transcriptionally profiled (Kurdyukov et al., 2014). Two of them expressed in zygotic embryos have been functionally characterized showing their essential roles during somatic embryogenesis. The first one from the ERF subfamily transcription factors, *SOMATIC EMBRYO RELATED FACTOR1 (MtSERF1)*, was showed to be induced by ethylene, in combination with other phytohormones, to control the initiation of somatic embryogenesis. Indeed, the *serf1* knock-down (KD) lines, obtained using RNAi, resulted in a strong suppression of somatic embryogenesis (Mantiri et al., 2008). Some evidence supported that *MtSERF* could be regulated by *WUSCHEL* gene (*MtWUS*). Indeed, *wus* RNAi lines displayed similar phenotype as *serf1*, with no formation of callus and somatic embryos, and WUS binding sites were identified in the *SERF1* promoter (Chen et al., 2009).

Apart from the regulation of the morphogenesis, embryogenesis is also marked by active cell divisions within the embryo, which is crucial for the final determination of the mature seed size. Indeed, a positive correlation was observed between the number of cotyledon cells and the final seed size in pea and soybean (Munier-Jolain and Ney, 1998). Two genes expressed in the endosperm of *M. truncatula* have been described as regulators of embryo cell division thereby impacting the seed size. The loss-of-function mutation in the *SBT1.1* gene, a subtilase, was obtained from an EMS population by TILLING screening and its phenotype showed reduced cell number and a decrease of seed size compared with wild type plants. This suggests that *SBT1.1* can regulate the cell number of cotyledon during the seed development, probably by providing molecules that act as signals to control cell division within the embryo (D'Erfurth et al., 2012). Moreover, a *DOF* transcription factor, *DASH* (*DOF Acting in Seed embryogenesis and Hormone accumulation*) was demonstrated to regulate the cell division rate of the embryo by its impact on auxin homeostasis leading to final seed size determination (Noguero et al., 2015). Three alleles were identified for *MtDASH, one* weak allele (i.e., *Tnt1* insertion in the promoter region) showing a moderate phenotype on seed size and two strong alleles (i.e., *Tnt1* insertion just before the starting codon and an EMS substitution inducing premature stop codon) displaying embryo lethal phenotypes and defect in auxin homeostasis.

### Seed composition

The seed composition represents a nutritionally important trait. Indeed, the nature and amount of stored macromolecules will define the final quality of seeds for human and animal diets. In *M. truncatula*, as in the dicot seeds, most of these storage macromolecules are stored in the embryo during the seed filling phase from 14 dap to 36 dap. To our knowledge, only one gene, *MtSAP1*, has been shown to directly regulate the nature and amount of the major storage molecules in *M. truncatula*. Using RNAi, *sap1* seeds displayed reduction of the globulin content (i.e., legumin and vicillin storage proteins). This phenotype affecting the seed composition was associated with reduced seed size and decrease of germination rate (Gimeno-Gilles et al., 2011). Other molecules, called secondary metabolites, are accumulated during the seed development, in another seed tissue, the seed coat. These molecules are not required for the proper seed development and are proposed to participate in defense-related processes. Amongst these compounds, the flavonoids such as proanthocyanindins (or PA) and anthocyanins have recently received a lot of attention because of their potential beneficial effects on human and animal health. To date, regarding the regulation of the PA biosynthetic pathway, several genes have been identified in *M. truncatula* such as *MtWD40-1* (Pang et al., 2009), *MtMYB5* and *MtMYB14* (Liu et al., 2014), *MtPAR* (*PROANTHOCYANIDIN REGULATOR, a* regulator of PA accumulation by its action on *MtWD40-1*; Verdier et al., 2012). All these regulator genes have been shown to directly or indirectly control one of the major enzymes leading to PA production, the ANTHOCYANIDIN REDUCTASE (ANR). Others genes with involvement in PA accumulation have been revealed, such as a glycosyltransferase gene (*UGT72L1*) involved in modification and assembly of PA precursors (Pang et al., 2013) and *MATE1*, a PA transporter (Zhao and Dixon, 2009). In parallel to the PA pathway, the anthocyanin biosynthetic pathway also received some attention. *MATE2* has been shown to transport the anthocyanin by redirecting the flavonoid precursors to the anthocyanin pathway at the expense of the PAs (Zhao et al., 2011). Moreover, the glycosyltransferase gene (*UGT78G1*) was found to be essential to anthocyanin modification and accumulation (Peel et al., 2009). Most of the advances in the PA/anthocyanin gene regulation have become possible using *Tnt1* insertion mutants of the previously mentioned genes.

### Desiccation tolerance

Desiccation tolerance represents an important seed quality trait that allows mature seeds to survive in a dry state for long periods of time. This characteristic is acquired at the later stages of maturation, after 20–24 dap in *M. truncatula* (Figure 2). Several protective molecular processes have been described to acquire the desiccation tolerance including accumulation of non-reducing sugars such as raffinose family oligosaccharides (RFO), late embryogenesis abundant *(LEA*) proteins and other stress-related proteins. In *M. truncatula*, several regulators of these processes have been identified. Knock-down of the *MtSNF4b* gene, which encodes the γ-subunit of *SnRK1* (sucrose non-fermenting-related kinase complex), resulted in impaired accumulation of RFO in seeds leading to a decrease of desiccation tolerance and seed longevity (Rosnoblet et al., 2007). Loss-of-function mutants of two other key regulators of seed development, *ABSCISIC ACID INSENSITIVE3* (*MtABI3*) and *ABSCISIC ACID INSENSITIVE5* (*MtABI5*), have been functionally characterized and revealed a loss of desiccation tolerance in mutant lines with impaired RFO content, decrease of LEA protein expression and changes in stress-related protein expression (Delahaie et al., 2013; Terrasson et al., 2013). Finally, *STAY GREEN* gene (*MtSGR*) have been identified and loss-of-function *sgr* mutants displayed a delayed senescence of the whole plant (Zhou et al., 2011). Even if the *sgr* mutant seed phenotype has not been investigated in the study, *sgr* mutant seed remained green at maturity, which mimics the loss of ability to desiccate of the *abi3* mutant seeds (Delahaie et al., 2013).

### Conclusion

In conclusion to this section, functional genetic studies of seed-related genes in *M. truncatula* are facing the same challenges than in other species: (i) the embryo and/or seed lethality phenotypes and (ii) the complexity of regulation between seed tissues with the seed coat (diploid and maternal origin, 2n♀), the endosperm (triploid and hybrid origin, 2n ♀ + 1n♂), and the embryo (1n♀ + 1n♂). Regarding the first challenge, *M. truncatula* mutant populations are kept and distributed at the segregating population status, which allows the conservation of most of the lethal mutations at the heterozygote status, when the mutation is non-dominant. However, this advantage of keeping living seeds containing 1 allele of lethal mutation, could turn as a time consuming process when it comes to identify homozygote mutant plants to reveal putative seed phenotype due to the *M. truncatula* generation time (~4 months). So far, most of the functional genetic studies have focused on the seed coat composition and flavonoid accumulation because it is a non-lethal mutation and easy to phenotypically visualize (i.e., change of seed coat color). This aspect represents an economical interest because of the beneficial effects of tannins on plant defense, flower color, forage quality and human health (see review, Dixon et al., 2013). The second focus of *M. truncatula* seed studies was the genes related to desiccation tolerance because we know how to rescue seeds non-tolerant to desiccation. At the opposite, to date, there is no efficient protocol in *M. truncatula* for embryo-rescue, which limits the functional studies of genes essential for proper embryogenesis. Regarding the second challenge, most of the genetic studies related to the complexity of regulation of seed development have been using *A. thaliana* as model plant. As demonstrated in the current section, *M. truncatula* seed genetic studies have so far focused on more applied aspects of seed biology such as yield, seed composition or desiccation/longevity.

## Functional genetics of abiotic stresses

Being sessile, plants have acquired the ability to respond promptly to extreme environmental conditions in order to survive. Among all the factors that affect plant growth, abiotic stresses probably have the largest effect. The primary abiotic stresses are drought, salinity, low and high temperature, nutrient deficiency, and flooding. Plants respond to each abiotic stress specifically but crosstalks among different stresses at physiological, biochemical, cellular, and molecular levels are common (Araujo et al., 2015). For example, both drought and salinity induce osmotic stress and therefore plants respond similarly to both stresses in osmotic-stress correlated pathways.

On large scales, microarray and RNA-seq experiments have been widely used to perform whole genome transcript profiling in *M. truncatula*. For example, identification of microRNAs responsive to drought (Wang et al., 2011), salinity (Long et al., 2015), and heavy metals (Zhou et al., 2011; Chen et al., 2012), and identification of gene expression changes under phosphate and nitrogen limitation (Bonneau et al., 2013), salinity stress (Gruber et al., 2009; Li et al., 2009), gradual drought stress (Zhang et al., 2014), as well as ozone stress (Puckette et al., 2008). At single gene level, functional studies of abiotic stress-related genes covered all the major aspects including gene expression (transcription factors), signaling transduction, osmolyte regulation, as well as antioxidant biosynthesis and DNA stability. Because of the crosstalk among stresses, researchers often identify plants' responses to multiple abiotic stresses in gene functional studies. Therefore, we classified the abiotic stress-related genes that have been functionally studied in *M. truncatula* based on functional category rather than the type(s) of abiotic stress (Table 3).

**Table 3:** **List of abiotic-stress related genes that have been functionally characterized in ***M. truncatula*****.

	**Stress category**	**Gene**	**Gene full name**	**Gene source**	**Approach**	**Promoter**	**Proposed gene function/description**	**References**
Transcription factor	Salinity	*Mtzpt2-1*	Kruppel-like zinc finger protein	Mtr	antisense transformation in Mtr	35S	Required for recovery from salinity stress in the roots	Merchan et al., 2003
	Salinity	*MtZpt2-1, MtZpt2-2*	Kruppel-like zinc finger protein	Mtr	over-expression in Mtr	35S	Promote root growth under salinity	de Lorenzo et al., 2007; Merchan et al., 2007
	Salinity, ABA, and osmotic	*HB1*	Homeobox 1	Mtr	Over-expression and TILLING mutants in Mtr	35S	Regulation of lateral root emergence under abiotic stresses	Ariel et al., 2010
	Freezing	*MtDREB1C*	Dehydration-responsive element binding	Mtr	overexpression in Mtr and rose	35S/Mtr; rd29A/rose	Enhancing freezing tolerance	Chen et al., 2010
	Drought and salinity	*MtCBF4*	C-repeat binding factor 4	Mtr	Over-expression in Arabidopsis and Mtr root	35S	Enhanced drought and salinity tolerance	Li et al., 2011
	Salinity	*MtNAC969*	(NAM/ATAF/CUC)-encoding	Mtr	Over-expression and RNAi in Mtr	35S	Inhibition of root growth and lateral root emergence under salinity	de Zélicourt et al., 2012
	Drought, salinity and freezing	*MtHB2*	Homeobox 2	Mtr	Over-expression in Arabidopsis	35S	Inhibition of osmolite accumuation, negative role in abiotic stress response	Song et al., 2012
Signaling	Salinity	*Srlk*	Salt-induced receptor-like kinase	Mtr	RNAi and TILLING mutants		Mediates root sodium update and early root response under salinity	de Lorenzo et al., 2009
	Drought and salinity	*MtCaMP1*	Calcium-binding motif-containing protein 1	Mtr	Over-expression in Arabidopsis	35S	Involved in osmo-regulation and anti-oxidation under stress	Wang T.-Z. et al., 2013
Osmolite	Salinity	*P5CS*	Delta-1-pyrroline-5-carboxylate synthetase	*Vigna aconitifolia*	Over-expression in Mtr	35S	Maintains nodule nitrogen-fixing activity under salinity stress	Verdoy et al., 2006
	Salinity	*MtP5CS3*	Delta-1-pyrroline-5-carboxylate synthetase	Mtr	RNAi and *Tnt1* mutants		Proline accumulation in the nodule under salinity stress	Kim and Nam, 2013
	Salinity, osmotic and drought	*MtP5CS3*	Delta-1-pyrroline-5-carboxylate synthetase	Mtr	*Tnt1* mutants and over-expression in Mtr	35S	Proline accumulation under stress, confers stress tolerance	Nguyen et al., 2013
Arial protection	Drought	*WXP1*	Wax production	Mtr	Over-expression in alfalfa	35S	Reduces water loss under drought	Zhang et al., 2005
	Drought and freezing	*WXP1 and WXP2*	Wax production	Mtr	Over-expression in Arabidopsis	35S	Reduces water loss under drought, but has either positive (WXP1) or negative (WXP2) roles under freezing stress	Zhang et al., 2007
Stress protein	Salinity and osmotic	*MtSAP1*	Stress associated proteins	Mtr	Overexpression in tobacco	35S	Promotes nitric oxide biosynthesis	Charrier et al., 2012
	Salinity, osmotic, cold, heat	*MtSAP1*	Stress associated proteins	Mtr	Overexpression in tobacco	35S	Promotes plant growth under abiotic stresses but not proline accumulation	Charrier et al., 2013
	Drought	*Dsp22*	Desiccation stress protein (22 kDa)	*Craterostigma plantagineum*	Over-expression in Mtr	35S	Assistance in recovery from water deprivation	Araujo et al., 2013
Miscellaneous	Anoxia	*AlaAT*	Alanine amino transferase		Non-transgenic		Involved in anoxia tolerance during seed germination	Ricoult et al., 2005, 2006
	Salinity	*flavodoxin*		*Anabaena variabilis*	Over-expression in Mtr	35S, plastid targeted	Maintains nitrogen-fixing activity under salinity but does not confer salinity tolerance to the entire plant	Coba de la Peña et al., 2010
	Osmotic and oxidative	*MtTdp2a*	Tyrosyl-DNA phosphodiesterase 2	Mtr	Over-expression in Mtr	35S, chl. targeted	Prevents accumulation of double strand breaks, enhance stress tolerance	Confalonieri et al., 2014
	Heavy metal (copper)	*MtTdp2a*	Tyrosyl-DNA phosphodiesterase 2	Mtr	Over-expression in Mtr	35S, chl. targeted	Prevents accumulation of double strand breaks, enhance stress tolerance	Fae et al., 2014
	Salinity	*MtCRE1*	Cytokinin response 1	Mtr	TILLING mutant		Inhibition of lateral root formation under both control and salinity stress	Laffont et al., 2015

Among all the **transcription factors** that have been characterized in *M. truncatula, Mtzpt2* (encoding a putative Krüppel-like Cys-2/His-2 zinc finger protein) has been extensively characterized. A homolog of *zpt2* was first identified in alfalfa (*Mscp17* and renamed *Mszpt2-1*) for its strong induction during nodulation (Frugier et al., 1998). Ectopic expression of *Mszpt2-1* and its Arabidopsis homolog, *Stz*, in yeast was able to induce salinity tolerance (Lippuner et al., 1996; Frugier et al., 2000). In *M. truncatula, Mtzpt2-1* is rapidly induced by salt treatment in both the nodules and the roots. Notably, antisense *Mtzpt2-1* transgenic *M. truncatula* plants had a significantly slower recovery compared to the control plants after salinity stress, although the root growth was similar before and during salinity stress between the transgenic and the control plants (Merchan et al., 2003). In an independent study, *Mtzpt2-1* and *Mtzpt2-2* were identified as among the most differentially regulated genes between *M. truncatula* genotypes Jemalong A17 and R108 under salinity stress, where Jemalong A17 was more tolerant to salinity than R108 (de Lorenzo et al., 2007). Further, overexpression of *Mtzpt2-1* and *Mtzpt2-2* in the salinity-sensitive genotype R108, but not in Jemalong A17, promoted root growth under salt stress (de Lorenzo et al., 2007). These results suggest a potential role of *Mtzpt2* in adaptation of *M. truncatula* to saline soils. Interestingly, at the same time period, Merchan et al. (2007) identified *Mtzpt2-1* and *Mtzpt2-2* as the top regulated genes in the R108 root during salt stress and recovery with a subtractive hybridization approach, and they revealed that overexpression of *Mtzpt2-1* in roots conferred plant salt tolerance by maintaining root growth. Taken together, *Mtzpt2* appears to be a strongly salinity-induced gene in the root and may confer protection to the root under salinity stress conditions.

Besides *Mtzpt2*, which has been studied in detail, *HB1, HB2, DREB1C, CBF4*, and *NAC969* have all been shown to be involved in gene expression regulation under various stress conditions in *M. truncatula* (Table 3). It is interesting to note that overexpression of *MtHB2* in Arabidopsis inhibited osmolyte accumulation rendering plants more sensitive to abiotic stresses (Song et al., 2012), while overexpression of *MtHB1* in *M. truncatula* altered root structure without negative effect on stress tolerance (Ariel et al., 2010).

Compared to transcription factors, genes involved in stress **signaling transduction** are less studied. In the same experiment as previously mentioned, Merchan et al. (2007) identified a *SRLK* (for Salt-induced Receptor-Like Kinase) gene. They showed that expression of *SRLK* was rapidly induced after salt stress in roots, especially in the root epidermis and root apex. *srlk*-TILLING mutants were able to maintain root growth under salt stress and also accumulated less sodium ions than the control plants in both root and shoot (de Lorenzo et al., 2009). Several early salt-regulated genes including *Mtzpt2-1* were down-regulated in the *srlk* mutant lines under salt stress, indicating that *SRLK* may function in the early steps of a salinity stress signal transduction pathway.

**Osmolyte** accumulation in plants under abiotic stress has been widely observed (reviewed in Ashraf and Foolad, 2007; Kavi Kishor and Sreenivasulu, 2014). The primary forms of osmolytes are proline, glycine betaine, sugars, and polyols. In *M. truncatula*, proline biosynthesis has been the focus of manipulation under abiotic stress. *P5CS* (*delta-1-pyrroline-5-carboxylate synthetase*), a gene encoding the major proline biosynthetic enzyme, has been studied with both loss- and gain-of function approaches. It was shown that under salinity stress, overexpression of *P5CS* gene in *M. truncatula* could maintain nodule nitrogen fixation activity under salinity stress (Verdoy et al., 2006) and the transgenic plant displayed increased tolerance to salinity and osmotic stresses (Nguyen et al., 2013). On the other hand, a loss-of-function mutant of *MtP5CS3* formed fewer nodules, and had lower nitrogen-fixation efficiency than the wild type under salinity (Kim and Nam, 2013). The non-nodulated *MtP5CS3* insertion-mutant plant (*Tnt1*-insertion mutant) accumulated less proline and showed increased sensitivity to drought, salinity, and osmotic stresses, resulting in decreased seedling growth and leaf chlorophyll content (Nguyen et al., 2013).

In addition to the above three major categories of abiotic stress-regulated genes, **genes that participate in other processes of plant abiotic stress responses** have also been explored in *M. truncatula*, though not extensively. *WXP1* and *WXP2* (*wax production protein*) are two homologous genes involved in the wax production pathway in *M. truncatula*. Overexpression of *WXP1* or *WXP2* in alfalfa and Arabidopsis significantly reduced water loss under drought and generated drought-resistant transgenic plants (Zhang et al., 2005, 2007). Interestingly, *WXP1* overexpression also promoted freezing tolerance, but *WXP2* overexpression had the opposite effect; though, the underlying mechanism has not been explained (Zhang et al., 2007). In addition, two other stress-related proteins were characterized. *MtSAP1* (*stress associated protein 1*) was over-expressed in tobacco and the transgenic plants performed better under multiple stresses with more nitric oxide production independently of enhanced proline accumulation (Charrier et al., 2012, 2013). Moreover, *DSP22* gene (*desiccation stress protein 22 kDa*) identified from the resurrection plant *Craterostigma plantagineum* was over-expressed in *M. truncatula* and the transgenic plants were able to recover from drought stress better than the wild-type plants (Araujo et al., 2013). Finally, other genes involved in amino acid production, ROS scavenging, DNA stability, as well as hormone signaling were functionally studied and are listed in Table 3.

### Conclusion

In conclusion to this section, if we analyze together the abiotic stress-related genes that have been functionally studied in *M. truncatula*, we see clear trends. First, salinity stress, rather than drought stress, has been the top focus among all abiotic stresses, due to the simplicity of controlling specific amount of salt in petri dishes experiments. Second, the majority of the functional genomics studies used over-expression approach, either alone or in combination with loss-of-function mutants. To generate overexpressing transgenic plants, the constitutive cauliflower mosaic virus (CaMV) 35S promoter was used in almost all studies except one (Chen et al., 2010). This potentially complicates the interpretation of transformed plant phenotypes considering the tissue-specific expression manner of the target genes. Finally, transgenic plants showing abiotic-stress tolerant phenotypes were only tested in the petri dish/growth chamber conditions, but not under soil and/or field conditions.

## Conclusion

In this review, we presented a non-exhaustive list of *M. truncatula* genes that have been functionally characterized in three primary research areas of legume biology. With the recent completion of the *M. truncatula* genome, the development of several genomics tools and the new technological advances, we have no doubt that we are only at the onset of functional genetics and genomics in this species and many other gene functions will be investigated and revealed in the coming years. For instance, transcriptomics studies have estimated that more than 20,000 plant genes are expressed during nodule development and more than 19,000 are differentially expressed during seed development in *M. truncatula*. Among them, only ~100 genes have been characterized functionally in different plant species. Extensive use of next generation technology coupled with genomics and systems biology approaches is needed for the better understanding of the functional aspects of the gene pool mentioned above. Finally, a technical breakthrough with the recent development of genome editing technologies, such as TALENs, ZINC-FINGER nucleases, and CRISPR-CAS9, provide new tools to precisely mutate gene sequences in order to advance functional genomics studies.

## Author contributions

All authors contributed equally to the work. YK, ML, SS and JV wrote the paper.

### Conflict of interest statement

The authors declare that the research was conducted in the absence of any commercial or financial relationships that could be construed as a potential conflict of interest.
